# Supplementary material for: Impact of the 2024 Korean medical workforce crisis on transfers in a pediatric emergency center: including comparative analyses with adults
Source: PLoS One. 2026 May 19;21(5):e0348189. doi: 10.1371/journal.pone.0348189 (PMC13186376; doi:10.1371/journal.pone.0348189)
Supplement: S1 Table — (DOCX) [file pone.0348189.s002.docx]

**S1 Table. Inverse probability of treatment weighting (IPTW)-adjusted logistic regression results for transfer outcomes in pediatric group.**

| **Variable** | **OR** | **95% CI** | **p-value** |
| --- | --- | --- | --- |
| **(Intercept)** | 0.00 | 0.00, 0.00 | **<0.001** |
| **Period (After vs Before)** | 2.21 | 1.55, 3.16 | **<0.001** |
| CI = Confidence Interval, OR = Odds Ratio | | | |
