# Supplementary figures and images for: Impact of the 2024 Korean medical workforce crisis on transfers in a pediatric emergency center: including comparative analyses with adults
Source: PLoS One. 2026 May 19;21(5):e0348189. doi: 10.1371/journal.pone.0348189 (PMC13186376; doi:10.1371/journal.pone.0348189)

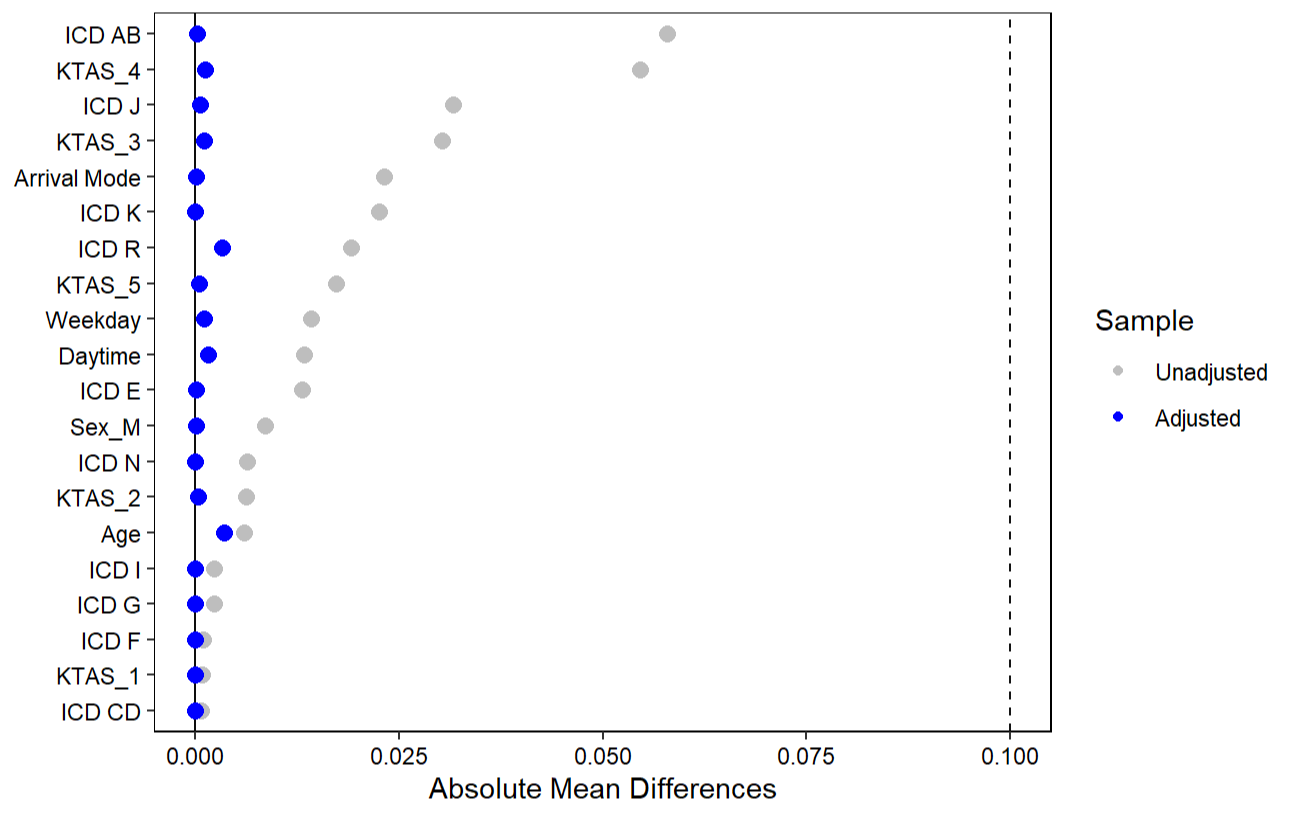

Supplement: S1 Fig — The plot displays standardized mean differences (SMDs) for baseline covariates comparing pre- and post-policy announcement periods. Gray dots indicate covariate balance before weighting, and blue dots indicate balance after IPTW adjustment. The vertical dashed line at an absolute SMD of 0.1 represents the conventional threshold for acceptable covariate balance. ICD, International Classification of Diseases; KTAS, Korean Triage and Acuity Scale. (TIFF) [file pone.0348189.s001.tiff]
