# Supplementary material for: Impact of the 2024 Korean medical workforce crisis on transfers in a pediatric emergency center: including comparative analyses with adults
Source: PLoS One. 2026 May 19;21(5):e0348189. doi: 10.1371/journal.pone.0348189 (PMC13186376; doi:10.1371/journal.pone.0348189)
Supplement: Attachment — Submitted filename: Response letter.docx [file pone.0348189.s004.docx]

**General Response**

We sincerely thank the Academic Editor and both reviewers for their thoughtful and constructive comments.

We carefully revised the manuscript to address all major and minor points.

**Key improvements include:**

Additional diagnostics and model refinements in the interrupted time series (ITS) analysis, including Durbin–Watson and Breusch–Godfrey tests, heteroskedasticity- and autocorrelation-consistent (HAC) standard errors, and generalized least squares (GLS) with AR(1) structure.

Sensitivity analyses excluding an influential outlier month (December 2024).

Clear specification of separate adult and pediatric logistic regression models, with both unadjusted and adjusted ORs presented in Table 3.

Re-organization of the inverse-probability-of-treatment-weighting (IPTW) analysis as a supporting verification rather than a main analysis.

Expanded Discussion addressing the methodological distinction between logistic and ITS approaches and updated Limitations acknowledging sample-size effects and single-center design.

Preparation of a de-identified aggregate dataset compliant with PLOS ONE’s data-availability policy.

All tracked revisions have been accepted, and both a clean and tracked version are provided.

Journal Requirements:

1. Please ensure that your manuscript meets PLOS ONE's style requirements, including those for file naming. The PLOS ONE style templates can be found at

**The manuscript follows PLOS ONE style and file-naming conventions.**

2. In the online submission form, you indicated that [The dataset analyzed in this study is not publicly available due to the policy of the institutional review board of Bundang CHA Medical Center. However, datasets are available from the corresponding author upon reasonable request.].

**Thank you for pointing out the need to update our Data Availability statement to comply with PLOS ONE requirements. We have now deposited the fully anonymized dataset, aggregated monthly transfer data, and accompanying README file in Zenodo. These files are publicly accessible at the following DOI:** [**https://doi.org/10.5281/zenodo.17823408**](https://doi.org/10.5281/zenodo.17823408)**.**

**We have also revised the Data Availability Statement to include a non-author, institutional point of contact. As required, requests for access to the original non-anonymized clinical data may now be directed to the Institutional Review Board (IRB) of Bundang CHA Medical Center at irb@chamc.co.kr, which serves as an independent institutional body responsible for evaluating data access requests in accordance with ethical regulations.**

3. Please include your full ethics statement in the ‘Methods’ section of your manuscript file. In your statement, please include the full name of the IRB or ethics committee who approved or waived your study, as well as whether or not you obtained informed written or verbal consent. If consent was waived for your study, please include this information in your statement as well.

**“Ethics Statement” is now included in the *Methods* section.**

1. Is the manuscript technically sound, and do the data support the conclusions?

The manuscript must describe a technically sound piece of scientific research with data that supports the conclusions. Experiments must have been conducted rigorously, with appropriate controls, replication, and sample sizes. The conclusions must be drawn appropriately based on the data presented.

Reviewer #1: Partly

Reviewer #2: Partly

**Thank you for your comment. I have prepared and uploaded a de-identified aggregate dataset in accordance with the journal’s data availability policy.**

2. Has the statistical analysis been performed appropriately and rigorously?

Reviewer #1: Yes

Reviewer #2: Yes

**Thank you for your comment. This retrospective study employed multivariable logistic regression, interrupted time series (ITS) analysis, and supplementary inverse probability of treatment weighting (IPTW), all of which were rigorously performed with appropriate model diagnostics and sensitivity analyses.**

3. Have the authors made all data underlying the findings in their manuscript fully available?

The PLOS Data policy requires authors to make all data underlying the findings described in their manuscript fully available without restriction, with rare exception (please refer to the Data Availability Statement in the manuscript PDF file). The data should be provided as part of the manuscript or its supporting information, or deposited to a public repository. For example, in addition to summary statistics, the data points behind means, medians and variance measures should be available. If there are restrictions on publicly sharing data—e.g. participant privacy or use of data from a third party—those must be specified.

Reviewer #1: No

Reviewer #2: Yes

**Thank you for pointing out the need to update our Data Availability statement to comply with PLOS ONE requirements. We have now deposited the fully anonymized dataset, aggregated monthly transfer data, and accompanying README file in Zenodo. These files are publicly accessible at the following DOI:** [**https://doi.org/10.5281/zenodo.17823408**](https://doi.org/10.5281/zenodo.17823408)**.**

**We have also revised the Data Availability Statement to include a non-author, institutional point of contact. As required, requests for access to the original non-anonymized clinical data may now be directed to the Institutional Review Board (IRB) of Bundang CHA Medical Center at irb@chamc.co.kr, which serves as an independent institutional body responsible for evaluating data access requests in accordance with ethical regulations.**

4. Is the manuscript presented in an intelligible fashion and written in standard English?

PLOS ONE does not copyedit accepted manuscripts, so the language in submitted articles must be clear, correct, and unambiguous. Any typographical or grammatical errors should be corrected at revision, so please note any specific errors here.

Reviewer #1: No

Reviewer #2: Yes

**Thank you for your comment. We carefully revised the manuscript to improve clarity and readability. All typographical and grammatical errors identified during the revision process have been corrected, and the text has been edited for standard academic English.**

5. Review Comments to the Author

Please use the space provided to explain your answers to the questions above. You may also include additional comments for the author, including concerns about dual publication, research ethics, or publication ethics. (Please upload your review as an attachment if it exceeds 20,000 characters)

**Point-by-point responses to reviewers' comments:**

Reviewer #1: The authors present 3 inter-related analyses of hospital transfers before and after a key policy change which led to an acute workforce shortage among South Korean physicians in February 2024. Interrupted time series did not find any significant change in pediatric transfers after the policy change (though there did appear to be a pre-existing upward trend); in contrast, multivariable logistic regression and IPTW did find that the post-policy time period seemed to be associated with an increase in transfer rate (allbeit no longer accounting for the pre-existing upward trend).

I think the work is overall an interesting opportunity to analyze the real world effects of a discrete policy change. It is both well suited to time series analysis and I think reasonable/appropriate to include at least one other analysis to adjust for potential changes in patient traits over time - though I think conducting both IPTW and multivariate regression is a bit duplicative as both adjust for this issue. The exploration of patterns of systemic disease categories pre and post policy change becomes quite speculative/exploratory, though at least p values were aggressively adjusted for (BH method given risk of false discovery).

I do think a number of areas could benefit from extra clarification/cleanup:

1) On selection of 3 different analytical methods: This work certainly seems best suited to interrupted time series analysis (which was one of the methods undertaken). I am a bit curious about the specific reasons for using both of the other methods. To me it would seem that multivariable logistic regression and IPTW effectively seek to accomplish similar goals (e.g., assessing likelihood of transfer by pre- or post-policy period while adjusting for potential confounders among patient-level characteristics).

-I think interrupted time series makes good sense as the primary analysis method

-I would next typically pick either multivariate logistic regression or IPTW (rather than both) as the second analysis - and frame it more clearly as an attempt to address/minimize potential confounding by differences in patient populations.

-If the authors really want to show the effect of potential confounders, then the multivariate regression approach might make the most sense as it more explicitly shows these effects; if the authors are most interested in simply looking at the overall trend in transfers (which seems to be their stated goal), then IPTW alone might make sense.

-If the authors really wanted to present results from both multivariate logistic regression and IPTW, I would still pick one or the other for the main manuscript and present the other as an alternative approach (perhaps in Supplemental materials) more as a sensitivity analysis showing that both come up with similar results. This would avoid making it seem as though the two are truly fully separate analyses when I don't see them as such (I suspect the IPTW weighting model actually closely resembles the logistic regression model).

-On that note, I was not clear what variables were utilized for IPTW - it would be worth specifying

**Authors’ Response: Thank you for your comment. We agree and have clarified that ITS was used as the primary approach to assess temporal changes, whereas logistic regression evaluated associations after adjusting for patient-level covariates. IPTW was retained only as a supporting verification, now moved to *Supporting Information.* The *Methods* section was revised accordingly.**

2) Appreciate the specific reference to assessment for auto-correlation and use of robust standard errors (lines 133-135), though the authors do not actually mention whether there was evidence of auto-correlation. If there were evidence of autocorrelation, there are usually additional modeling steps needed to account for that.

**Authors’ Response: Thank you for this comment. We added explicit results of Durbin–Watson and Breusch–Godfrey tests. The latter suggested AR(1) correlation in the adult series; therefore, GLS models with AR(1) structure and HAC (Newey–West) standard errors were applied. These revisions appear in *Methods* and *Results* sections. Figure 2 and Table 4B were updated to reflect autocorrelation-adjusted estimates.**

3) Line 137: Would soften/avoid language such as "To verify causal inference" as no adjustment can ever fully verify causal inference. Might suggest alternative language such as "To mitigate or address potential confounding by differences in patient populations pre- and post-policy announcement..."

**Authors’ Response: Thank you for pointing out the overly strong phrasing and suggesting a clearer alternative for it, we revised to “** **To mitigate potential confounding by differences in patient populations pre- and post-policy announcement, we additionally performed IPTW”.**

4) Table 2: The presentation of Table 2 makes me begin to wonder how age grouping was handled (Adult vs Pediatric) - were two separate models constructed for each age group? Or was age group considered as a variable?

-If part of the point of the article were that adult and pediatric trends differed, one would usually consider something like an interaction term to statistically test whether adult/pediatric trends differed. This would usually provide justification for producing separate models.

-Regardless of how age group were handled, many statistically inclined readers might like to see separate columns displaying the univariate (unadjusted) and multivariate (adjusted) ORs as this would help the reader understand the effect of adjustment for suspected confounders.

**Authors’ Response: We agree that clarification on separate models is needed. Separate multivariable logistic regressions were conducted for adult and pediatric groups.**

**Also, table 3 now includes both unadjusted and adjusted ORs (95 %CIs) for each variable, including detailed breakdowns by initial KTAS score (1–5).**

**The *Methods* section now specifies the modeling approach.**

5) Table 3A: I would suggest only showing the adjusted p values as these are more exploratory/subgroup based analyses.

**Authors’ Response: Thank you for the comment, unadjusted p values were removed; only Benjamini–Hochberg-adjusted p values now remain.**

6) Table 4: similar to above, I am curious if two separate models (adult and pediatric) were set a priori or if there were specific interest in demonstrating their trends differed (in which case again an interaction test might help assess whether these are statistically significantly different from one another).

**Authors’ Response: We greatly appreciate this insightful suggestion. We believe this specific review gave us a chance to widen our perspective in analyzing and proving the difference between the trends from different groups. A pooled logistic model with a *period × age-group* interaction term was added. The interaction was significant (OR 2.44, 95 % CI 1.56–3.85, p < 0.001), indicating a differential post-policy effect between adults and children. Results are described in the *Methods* and *Results* sections.**

7) Figure 2: There is a single month near what appears to be "2025-12" with a near 1.2% transfer rate. As this point at least visually looks as though it could serve as an influential outlier, consider sensitivity analysis of trend with exclusion of this point.

**Authors’ Response: Thank you for this comment. After the review, authors agree that sensitivity analysis would be more beneficial to understand the overall trend. We re-estimated ITS models excluding December 2024. The direction and significance of results were unchanged, confirming robustness. These are presented in *Results* and *Figure 2.***

8) In discussion of results, some of the above suggestions regarding methods might also apply:

-As expected, it seems IPTW and multivariate logistic regression came to the same conclusion that rate of pediatric transfers increased post policy change. This would seem to support that these are not really fully independent analyses (as in comment 1 above), but perhaps ought to have only one presented as a main analysis and the other simply as verification in Supplement.

-Stating that if the study period were only longer the difference would have reached significance seems rather speculative - suggest softening this language (lines 294-297).

-Probably worth a more nuanced discussion of the disparate findings between overall ITS and the multivariate adjusted analysis. Conceptually, ITS does better at adjusting for pre-existing trends and I think this seems to be part of the lack of significant findings when looking at Figure 2 - since there was already a pre-policy trend towards increasing pediatric transfers. This is likely worth acknowledging as a caveat.

-Getting back to my earlier question about testing for difference in trends between adults and pediatrics, I do still find myself wondering whether the interaction test for difference in post-policy change trends would suggest a difference since the adult pattern leveled off but pediatrics did not. This result at least would be more consistent with the difference noted in the multivariate analysis.

-The authors may consider noting that because the multivariate analysis did not abrogate the post-policy association with pediatric transfers, they do at least have some evidence the increase over time is not solely due to an changing demographics/conditions.

**Authors’ Response: Thank you for this comment. The following revisions have been made to address these points:**

**ITS vs logistic regression nuance: Added paragraph explaining that ITS accounts for pre-existing trends and autocorrelation, which attenuated the apparent post-policy effect, yielding more conservative results.**

**Persistence after adjustment: Expanded to note that the post-policy increase in pediatric transfers remained significant after adjustment, suggesting that this pattern is not solely explained by demographic or clinical shifts.**

**In accordance with the reviewer’s suggestions, we made several revisions in the Discussion. First, language implying that a longer study period “might have led to statistical significance” has been softened to avoid speculative interpretation.**

9) On data availability: Given PLOS One's data availability clause, are at least select aggregate data available for upload? E.g., surely numerator/denominator of transfers each month by adult and pediatric patients could be made available as this has no direct tie to any identifiers?

**Authors’ Response: Thank you for pointing this out. We have now deposited the fully anonymized dataset, aggregated monthly transfer data, and accompanying README file in Zenodo. These files are publicly accessible at the following DOI:** [**https://doi.org/10.5281/zenodo.17823408**](https://doi.org/10.5281/zenodo.17823408)**.**

Minor comments:

1) Abstract - results: would avoid use of "increase" or "decrease" unless statistically significant. Might instead prefer simply stating the number of transfers for each period.

**Authors’ Response: Thank you for this comment. We revised wording to describe counts rather than “increase/decrease” where statistical significance was absent.**

2) Introduction - line 55: Was this meant to read "In 2024" rather than "In 2004"?

**Authors’ Response: We corrected “2004” → “2024”.**

3) There are several areas where wording could be edited for clarity/brevity:

- Lines 64-66

- Line 71

**Authors’ Response: Thank you for pointing this out. We edited those lines for clarity and brevity**

4) Table 1:

-Defer to the Journal's specific statistical style recommendations, but I would usually refrain from reporting p values for baseline variable tables (to avoid false impression that these are hypothesis driven tests). These could either be simply omitted, or if authors feel it is important to display a measure of imbalance, standardized mean differences might provide the necessary information while avoiding the use of p values in a demographics table.

-Suggest reporting by each individual KTAS score rather than lumping 1-3 and 4-5 unless there is clear justification for this grouping

**Authors’ Response: Thank you for this helpful comment. Following the reviewer’s suggestion, all p-values have been removed from Table 1. KTAS scores are now presented individually (1–5) instead of being grouped.**

Reviewer #2: Thank you for conducting this research. I have a few questions about gaps that were not addressed in the study.

You briefly mentioned that there was huge decrease between the number of patients from pre-policy to post-policy. Does the change in sample size affect the statistical tests? You briefly discussed limitations of the study in the discussion session. Secondly, you mentioned that the study was conducted in one pediatric emergency center. Where there other events or policy changes that might contributed to the reduction in sample size. Did you consider other factors such as secular trends or other historial events that might have contributed to increase in pediatric transfers or decrease in junior doctors?

**Authors’ Response: We appreciate the reviewer’s insightful comment regarding the potential effect of the sample size reduction between periods on the statistical inference. In the interrupted time series (ITS) analysis, the monthly number of visits (denominator) directly contributes to the variance structure through the quasibinomial model, thereby accounting for varying sample sizes across time points. In addition, the generalized least squares (GLS) models with AR(1) correlation and the HAC-robust estimators further adjusted the standard errors for temporal dependence and heteroskedasticity. These procedures collectively ensure that the uncertainty associated with months having smaller denominators is reflected in the wider confidence intervals and more conservative inference. The consistent direction of estimates and nonsignificant slope changes, even after sensitivity analysis excluding an outlier month, suggest that the findings are robust and not unduly driven by sample size differences.**

**Nonetheless, we acknowledge that the ITS framework cannot fully eliminate the influence of reduced sample size on statistical power or residual variance. This limitation has been explicitly added to the revised manuscript, noting that although monthly weighting mitigates much of the imbalance, the smaller post-policy denominator may still contribute to wider confidence intervals and less precise estimates. We also acknowledge that unmeasured contextual factors could have contributed to patient-flow changes, which remain a limitation and now being addressed in the *Discussion* section.**

We are grateful for the reviewers’ constructive feedback, which has greatly improved the clarity, rigor, and transparency of our work.
We believe that the revised manuscript now fully addresses all reviewer and editorial concerns and meets PLOS ONE’s standards for publication.
