# Supplementary material for: Impact of the 2024 Korean medical workforce crisis on transfers in a pediatric emergency center: including comparative analyses with adults
Source: PLoS One. 2026 May 19;21(5):e0348189. doi: 10.1371/journal.pone.0348189 (PMC13186376; doi:10.1371/journal.pone.0348189)
Supplement: Attachment — Submitted filename: Response_letter_auresp_2.docx [file pone.0348189.s005.docx]

**General Response**

**We sincerely thank the Academic Editor and the reviewer for their careful re-evaluation of our revised manuscript and for acknowledging the additional analyses and clarifications provided in response to the previous round of review. We greatly appreciate the positive assessment that our additional work has adequately addressed all prior concerns, and we are grateful for the constructive and thoughtful feedback that helped improve the clarity and presentation of the manuscript.**

Reviewers' comments:

Reviewer's Responses to Questions

Comments to the Author

1. If the authors have adequately addressed your comments raised in a previous round of review and you feel that this manuscript is now acceptable for publication, you may indicate that here to bypass the “Comments to the Author” section, enter your conflict of interest statement in the “Confidential to Editor” section, and submit your "Accept" recommendation.

Reviewer #1: All comments have been addressed

2. Is the manuscript technically sound, and do the data support the conclusions?

The manuscript must describe a technically sound piece of scientific research with data that supports the conclusions. Experiments must have been conducted rigorously, with appropriate controls, replication, and sample sizes. The conclusions must be drawn appropriately based on the data presented.

Reviewer #1: Yes

3. Has the statistical analysis been performed appropriately and rigorously?

Reviewer #1: Yes

4. Have the authors made all data underlying the findings in their manuscript fully available? The PLOS Data policy requires authors to make all data underlying the findings described in their manuscript fully available without restriction, with rare exception (please refer to the Data Availability Statement in the manuscript PDF file). The data should be provided as part of the manuscript or its supporting information, or deposited to a public repository. For example, in addition to summary statistics, the data points behind means, medians and variance measures should be available. If there are restrictions on publicly sharing data—e.g. participant privacy or use of data from a third party—those must be specified.

Reviewer #1: Yes

5. Is the manuscript presented in an intelligible fashion and written in standard English? PLOS ONE does not copyedit accepted manuscripts, so the language in submitted articles must be clear, correct, and unambiguous. Any typographical or grammatical errors should be corrected at revision, so please note any specific errors here.

Reviewer #1: Yes

6. Review Comments to the Author Please use the space provided to explain your answers to the questions above. You may also include additional comments for the author, including concerns about dual publication, research ethics, or publication ethics. (Please upload your review as an attachment if it exceeds 20,000 characters)

Reviewer #1: I appreciate the extensive extra work the authors undertook in response to my questions. All of my questions have been fully answered. Only one minor consideration remains: since the responses make clear that ITS was the primary analysis method, would consider modifying current abstract (and perhaps some of discussion) to lead with the ITS results & discuss the other regression approaches secondarily (which seems to match the analysis plan a bit more closely).

**Thank you for your comment. In response to the reviewer’s suggestion, we revised the abstract to lead with the interrupted time series (ITS) results, with regression-based analyses presented subsequently. No changes were made to the underlying analyses or conclusions.**
