# Supplementary material for: Impact of the 2024 Korean medical workforce crisis on transfers in a pediatric emergency center: including comparative analyses with adults
Source: PLoS One. 2026 May 19;21(5):e0348189. doi: 10.1371/journal.pone.0348189 (PMC13186376; doi:10.1371/journal.pone.0348189)
Supplement: Attachment — Submitted filename: Response_Letter_auresp_3.docx [file pone.0348189.s006.docx]

**General Response**

**We sincerely thank the Academic Editor and the reviewer for their careful re-evaluation of our revised manuscript.**

**We greatly appreciate the positive assessment that our additional work has adequately addressed all prior concerns, and we are grateful for the constructive and thoughtful feedback that helped improve the clarity and presentation of the manuscript.**

Reviewers' comments:

1. Reviewer #1: Thank you for updating the abstract - I believe it now better reflects the hard work done, and all of my comments have been answered.

**We greatly appreciate the positive assessment.**

Reviewer #3: Dear Authors,

Thank you for submitting your manuscript entitled “Impact of the 2024 South Korean Junior Doctor Workforce Departure on Interhospital Transfers from a Pediatric Emergency Center: A Comparative Analysis with Adult Patients.” This is a timely and policy-relevant study examining the impact of the medical school quota expansion announcement and subsequent junior doctor resignations on emergency care, with a useful pediatric–adult comparison.

The topic is important, and the analytical approach, interrupted time series supplemented by multivariable regression and IPTW, is appropriate. The manuscript is generally well structured and offers relevant insight into pediatric vulnerability. However, several points need clarification to improve rigor and interpretability before publication.

1. The pre- (March 2023 to January 2024) and post-policy (March 2024 to January 2025) periods are appropriate for seasonality control. Please specify the exact number of months included in each period and confirm data completeness through January 2025.

**Thank you for this comment. We have added a sentence to the Methods section explicitly confirming the study period duration and data completeness: “Each study period therefore comprised 11 months (March through January), and data collection was confirmed to be complete through January 2025.”**

2. For the ITS analysis, clarify whether counts or rates were modeled, how inverse-variance weighting was implemented, and how multiple ICD-10 diagnoses per patient were handled.

**Thank you for this comment. We have revised the Statistical Analysis section to address these clarifications. First, the phrase “monthly counts of ED visits and transfers” was corrected to “monthly transfer rates (modeled as the proportion of transfers among total ED visits, using a quasibinomial generalized linear model).” Second, inverse-variance weighting (1/n_visits) was already described in the context of the GLS sensitivity analysis; no additional change was required. Third, we added an explicit description of how multiple ICD-10 diagnoses were handled.**

3. The rationale for excluding R codes in subgroup analysis should also be stated more clearly.

**Thank you for this comment. We have added explicit rationale for the exclusion of R codes to the Methods section.**

4. There is a discrepancy between the non-significant ITS findings and the significant associations observed in the regression and IPTW analyses for pediatric transfers (adjusted OR approximately 2.49 and 2.21). While this is acknowledged, it should be more explicitly framed in terms of methodological differences.

**Thank you for this important comment. We have substantially revised the corresponding paragraph in the Discussion to provide an explicit methodological explanation. Specifically, we clarified that ITS operates at the population level, modeling monthly aggregated transfer rates with only 11 data points per group, limiting statistical power for detecting modest changes; additionally, the substantial decline in pediatric ED visit volume (approximately 42%) introduces a denominator effect that the ITS model partially absorbs. In contrast, multivariable logistic regression and IPTW operate at the patient level and directly isolate the association between the period indicator and individual transfer probability after adjusting for case-mix confounders. The directional consistency across all three analytical approaches was highlighted as supporting the overall interpretation that the post-policy period was associated with a genuine increase in pediatric transfer risk.**

5. The increase in pediatric transfers (48 to 89) despite a roughly 42 percent decline in visits is a key result and should be presented more prominently across the abstract, results, and conclusion.

**Thank you for this comment. We agree that this contrast is a key finding of our study. We have added explicit reporting of the absolute numbers and visit volume decline in three locations.**

6. Please also review the unadjusted ORs in Table 3, particularly for KTAS categories, for any inconsistencies.

**Thank you for this comment. We carefully reviewed all unadjusted and adjusted ORs for KTAS categories in Table 3 and confirmed they are internally consistent and monotonically decreasing across KTAS levels for both adult and pediatric groups, with KTAS 1 (most severe) serving as the reference category and higher KTAS levels (lower acuity) showing progressively lower odds of transfer. No numerical inconsistencies were identified. To improve clarity in the Results text, we revised the phrase “less severe or acute” to “with KTAS 1 as the most severe reference category, such that lower acuity levels showed substantially reduced odds of transfer.”**

7. The discussion appropriately highlights pediatric vulnerability, shifts in transfer reasons, and the added burden of specialist accompaniment. A brief expansion on plausible mechanisms, such as more risk-averse decision-making or limited pediatric readiness in general emergency departments, would strengthen interpretation while maintaining caution around causality. A short comparison with existing work on the 2024 crisis would also help contextualize the findings.

**Thank you for this constructive suggestion. We have expanded the Discussion. First, we elaborated on plausible mechanisms by referencing existing literature on heightened risk-averse decision-making during workforce shortages, the limited readiness of general emergency departments to provide specialized pediatric care, and the inherently higher complexity of pediatric diagnosis and management. Second, we added a comparison with a recently published nationwide interrupted time series study, which similarly documented a sharp, sustained decline in ED visits following the junior physicians’ walkout.**

8. The limitations section may be slightly condensed, and a brief note on broader policy implications for maintaining pediatric emergency readiness would add value.

**Thank you for these comments. Regarding the limitations we condensed it with more concise sentences to reduce redundancy. Regarding policy implications: we added a dedicated paragraph to the Discussion presenting the broader policy implications of our findings. This paragraph emphasizes that abrupt and large-scale workforce policy changes without adequate transition planning risk creating disproportionate disruptions in vulnerable subgroups such as pediatric patients, and calls for minimum staffing thresholds, pre-defined transfer protocols, and the designation of pediatric emergency capacity as a protected resource during workforce crises.**

Overall, this is a relevant and potentially impactful study. Addressing these points will improve clarity and strengthen the manuscript.

**Thank you for your comment. In response to the reviewer’s suggestion, we revised the manuscript.**
